# Cumulative defects in DNA repair pathways drive the PARP inhibitor response in high-grade serous epithelial ovarian cancer cell lines

**DOI:** 10.18632/oncotarget.10308

**Published:** 2016-06-27

**Authors:** Hubert Fleury, Euridice Carmona, Vincent G. Morin, Liliane Meunier, Jean-Yves Masson, Patricia N. Tonin, Diane Provencher, Anne-Marie Mes-Masson

**Affiliations:** ^1^ Centre de Recherche du Centre Hospitalier de l’Université de Montréal (CRCHUM), Montreal, Canada; ^2^ Institut du cancer de Montréal, Montreal, Canada; ^3^ Genome Stability Laboratory, CHU Research Center, Québec City, Canada; ^4^ Department of Molecular Biology, Medical Biochemistry and Pathology, Laval University Cancer Research Center, Québec City, Canada; ^5^ Cancer Research Program (CRP), The Research Institute of the McGill University Health Centre, Montreal, Canada; ^6^ Department of Human Genetics, McGill University, Montreal, Canada; ^7^ Department of Medicine, McGill University, Montreal, Canada; ^8^ Division of Gynecologic Oncology, Université de Montréal, Montreal, Canada; ^9^ Department of Medicine, Université de Montréal, Montreal, Canada

**Keywords:** olaparib, high-grade serous epithelial ovarian cancer, DNA repair pathways, NER, MMR

## Abstract

PARP inhibitors (PARPi), such as Olaparib, have shown promising results in high-grade serous (HGS) epithelial ovarian cancer (EOC) treatment. PARPi sensitivity has been mainly associated with homologous recombination (HR) deficiency, but clinical trials have shown that predicting actual patient response is complex. Here, we investigated gene expression microarray, HR functionality and Olaparib sensitivity of 18 different HGS EOC cell lines and demonstrate that PARPi sensitivity is not only associated with HR defects. Gene target validation show that down regulation of genes in the nucleotide excision repair (NER) and mismatch repair (MMR) pathways (*ERCC8* and *MLH1*, respectively) increases PARPi response. The highest sensitivity was observed when genes in both the HR and either NER or MMR pathways were concomitantly down regulated. Using clinical samples, patients with these concurrent down regulations could be identified. Based on these results, a novel model to predict PARPi sensitivity is herein proposed. This model implies that the extreme responders identified in clinical trials have deficiencies in HR and either NER or MMR.

## INTRODUCTION

Ovarian cancer is the most lethal of all gynecologic malignancies in North America [1]. This is attributed to the asymptomatic nature of the disease, resulting in a late stage diagnosis with a five-year survival rate of 45% [1]. The most common form is epithelial cancer of the ovary or fallopian tube (EOC), where approximately 70% of EOC patients present with a high-grade serous (HGS) histotype [2]. The etiology of EOC is unknown, although 15% are attributed to inherited genetic factors such as mutations in *BRCA1* and *BRCA2* which significantly increases risk [3]. Over the past 45 years, advances in surgery and chemotherapy have had little impact on overall patient survival [4, 5] underscoring the need for a greater understanding of the molecular basis of this disease and the development of new clinical tools for the detection and management of EOC patients.

Standard first line therapy of EOC consists of tumor cytoreductive surgery and treatment with platinum DNA alkylating agents such as carboplatin or cisplatin combined with the microtubule poison paclitaxel [5]. Although initial response rates are high (>70%), the disease eventually recurs in most patients who will develop chemoresistance [4, 5]. Several adjuvant drugs have been developed to improve EOC survival and decrease chemoresistance [6]. One area involves the poly (ADP-ribose) polymerase inhibitors (PARPi) such as Olaparib, Rucaparib, Veliparib, Niraparib, and BMN-673 [7–9]. PARPi were first introduced to treat breast cancer patients harboring germline *BRCA1*/*BRCA2* mutations based on the synthetic lethality context, where it has been proposed that a defect in one repair pathway is compatible with cell viability but results in cell death when combined with another repair pathway defect or inhibition [10]. BRCA1/2 plays a role in DNA repair by homologous recombination (HR) [11] and defects in BRCA1/2 contribute to loss or dysfunction of HR. Several models have been proposed to explain the synthetic lethality of HR-deficient cells to the PARPi, however due to the complex role of the PARP1 polymerase in repairing single and double strand DNA breaks, the complete mechanism is still not understood [8, 9].

In clinical trials, treatment with Olaparib as a single agent was promising in EOC patients as compared to triple negative breast cancer patients [12–14], and responses around 45% and 25% are observed in EOC patients with and without *BRCA1/2* mutations, respectively. The response observed in women with EOC lacking *BRCA1/2* mutations was attributed to ‘BRCA-ness’, a molecular genetic signature in cancers equivalent to those with a *BRCA1/2* mutation [15] where other HR components were deficient by mutation or were epigenetically silenced [16, 17]. It was recently shown that ~40% of HGS EOCs exhibit HR abnormalities [18], and measurement of HR function in primary cultures of EOC ascites correlated with *in vitro* Rucaparib response [19, 20]. The recent approval by the US Food and Drug Administration of Olaparib as maintenance therapy for platinum-sensitive BRCA-mutated HGS EOC patients [21], further highlights the importance of this class of drugs in EOC clinical management. However, the molecular features that would predict the response to such drugs is still largely unknown, as not all patients with BRCA-ness HGS EOCs respond to these drugs [12–14]. We postulate that defects in DNA repair pathways other than the HR are also involved in PARPi sensitivity. Having a molecular gene signature linked to PARPi sensitivity would help the selection of patients that will undergo such treatment and increase effectiveness.

To identify DNA repair genes associated with the PARPi response, we applied gene expression microarray analysis to our unique repertoire of 18 spontaneously immortalized HGS EOC cell lines [22–25]. DNA repair genes that were associated with PARPi sensitivity were validated by small interference RNA (siRNA) and analyzed in clinical samples. Although previous reports have described DNA repair genes as potential biomarkers for PARPi response [26–28], the function of these genes were predominantly related to the HR system. Here we demonstrate that highest PARPi sensitivity is achieved when HR deficiency is combined with a defect in the DNA mismatch repair (MMR) or nucleotide excision repair (NER) pathway, and we propose a novel model to predict PARPi sensitivity based on these results.

## RESULTS

### HGS EOC cell lines can be distinguished into three groups of Olaparib sensitivity

**Figure 1:**
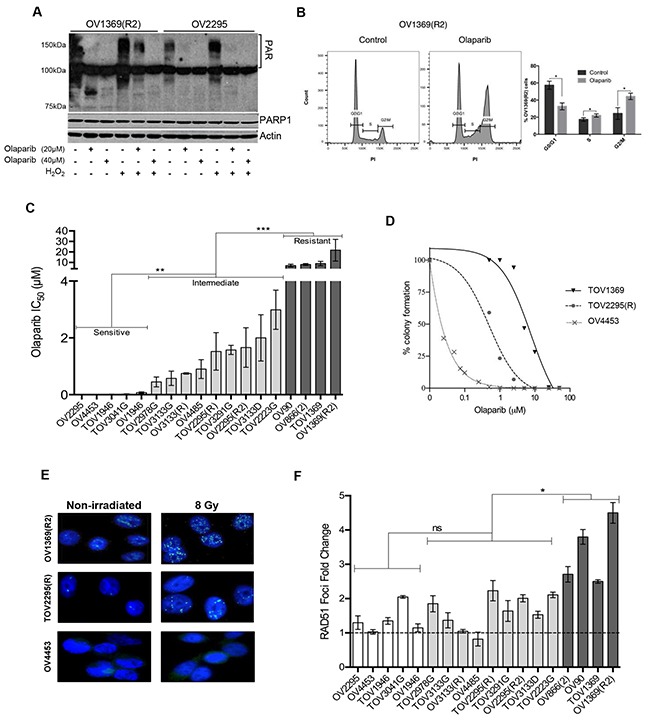
Analysis of Olaparib sensitivity and HR functionality in HGS EOC cell lines **A**. Western blot detection of PAR polymer and PARP1 in OV1369(R2) and OV2295 after a 24 hour exposure to 20 or 40 µM of Olaparib, with or without H_2_O_2_ pre-treatment (1 mM). Actin was used as a loading control. **B**. Flow cytometry analysis of cell cycle populations following exposure of OV1369(R2) cells to Olaparib (40 µM, 24 hours). Control = non-treated. Bars represent average ± SEM of percent cells in G0/G1, S, G2/M phases in control (dark grey) or Olaparib treated (light grey) cells obtained by three independent analyses. **C**. Olaparib sensitivity evaluated by clonogenic assays using different drug concentrations. Cell lines were categorized as Olaparib sensitive (white bars), intermediate (light grey bars) or resistant (dark grey bars). Bars represent average ± SEM of IC_50_ values obtained by three independent clonogenic assays. **D**. Olaparib sensitivity curves of cell lines representing each group: sensitive (OV4453), intermediate [TOV2295(R)] and resistant [OV1369(R2)]. **E**. Representative images of RAD51 immunostaining after 8 Gy irradiation compared with non-irradiated controls of three selected cell line: sensitive (OV4453), intermediate [TOV2295(R)] and resistant OV1369(R2). Images are at 400 X magnification. **F**. HR response evaluation by RAD51 immunocytochemistry. Nuclear foci were counted 2 hours after exposure to 8 Gy gamma-radiation, and then compared and presented as percentage of the control group (non-irradiated). Fold change was calculated as a ratio between percentages of Rad51 foci in treated over control non-treated cells. Bars represent average ± SEM and bar colors are as in Figure 1C. * denotes *p* < 0.05, ** *p* < 0.01, *** *p* < 0.001, ns = non-significant.

To better understand the PARPi response in HGS EOC, we used our unique collection of 18 HGS EOC cell lines derived from malignant tumors (TOV-) and ascites (OV-). These spontaneously immortalized cell lines have been extensively characterized [22–25]. Among the 18 cell lines, 17 harbor *TP53* mutations, which is the most common somatically mutated gene found in HGS EOCs, while the remaining line fails to express *TP53*. The only two cell lines with a germline *BRCA1* (OV4485) or *BRCA2* (OV4453) mutation [22] were used as positive controls for HR deficiency. As a background work, we confirmed the inhibitory activity of Olaparib on PARP (Figure 1) in a carboplatin resistant [OV1369(R2)] and a sensitive (OV2295) cell line [23], based on the knowledge that carboplatin and PARPi sensitivities correlate [13, 29]. Formation of poly (ADP-ribose) (PAR) polymers, which results from PARP enzymatic action, was detected by Western blot (Figure 1A). Olaparib significantly diminished PARP catalytic activity in both of these cell lines, in comparison to an untreated control or an H_2_0_2_-treated positive control where treatment of H_2_0_2_ induces DNA damage that is known to be repaired by PARP [30]. To verify the known effect of PARPi on cell cycle arrest [31], we treated OV1369(R2) cells with Olaparib for 24 hours and performed flow cytometry. When compared to the untreated cells (control), Olaparib-treated cells had significantly increased proportion of cells in G2/M and S phase, indicating the expected cell cycle arrest in G2 (Figure 1B).

We then examined the IC_50_ of Olaparib in our cell lines by clonogenic assay (Figure 1C–1D) and determined levels of sensitivity. Olaparib elicited a varied response with IC_50_ values that ranged from very low at 0.0003 μM (OV2295) to extremely high at 21.7μM [OV1369(R2)] (Figure 1C–1D, Table 1). This range of IC_50_ concentrations has been observed by others in ovarian cancer cell lines treated with Olaparib [16, 26]. Based on statistical analysis of the IC_50_ values (Figure S1A-B), the cell lines were classified into three distinct groups: sensitive (0.0003-0.074 μM), intermediate (0.4-3.0 μM) and resistant (7.0-21.7 μM). There were at least four cell lines within each grouping. Figure 1D shows examples of Olaparib dose-response curves for cell lines belonging to each group, and a log difference can be readily observed among them. As predicted, the *BRCA2* mutated cell line (OV4453) was classified as sensitive with an IC_50_ of 0.01 μM. However, OV4485, the cell line harboring a *BRCA1* mutation, had an IC_50_ that was classified as intermediate (Figure 1C). This suggests that *BRCA* mutations, and therefore defects in HR, are not solely responsible for conferring PARPi sensitivity to cancer cells.

**Table 1:** Quantification of Olaparib sensitivities (IC_50_) and RAD51 foci in EOC cell lines

		IC_50_ Olaparib (µM)	RAD51 foci fold change
**Resistant**	**OV1369 (R2)**	21.71 ± 10.33	4.5 ± 0.30
	**TOV1369**	9.02 ± 3.66	2.0 ± 0.05
	**OV866(2)**	8.11 ± 1.27	2.71 ± 0.21
	**OV90**	7.04 ± 2.33	3.80 ± 0.23
**Intermediate**	**TOV2223G**	2.99 ± 1.20	2.11 ± 0.08
	**TOV3133D**	2.00 ± 1.15	1.53 ± 0.1
	**OV2295(R2)**	1.66 ± 0.99	2.01 ± 0.1
	**TOV3291G**	1.58 ± 0.23	1.64 ± 0.3
	**TOV2295(R)**	1.52 ± 1.14	2.23 ± 0.29
	**OV4485**	0.90 ± 0.58	0.82 ± 0.2
	**OV3133(R)**	0.75 ± 0.04	1.05 ± 0.05
	**TOV3133G**	0.58 ± 0.44	1.37 ± 0.22
	**TOV2978G**	0.45 ± 0.30	1.85 ± 0.23
**Sensitive**	**OV1946**	0.07 ± 0.05	1.15 ± 0.11
	**TOV3041G**	0.02 ± 0.01	2.05 ± 0.04
	**TOV1946**	0.02 ± 0.007	1.35 ± 0.098
	**OV4453**	0.01 ± 0.0009	1.03 ± 0.067
	**OV2295**	0.0003 ± 0.0004	1.30 ± 0.2

### Defective HR is not the only predictor of Olaparib sensitivity in HGS EOC cell lines

Reports have described a direct association between the PARPi response and non-functional HR pathway [19, 32] such that deficiency in genes implicated in HR function (such as *BRCA1/2, RAD51C, PTEN*), has been shown to increase PARPi sensitivity [27, 33, 34]. Furthermore, deregulation of HR genes frequently occurs in HGS tumors [18]. Therefore, we examined the HR status of our cell lines by quantifying the increase in mean nuclear RAD51 foci after DNA damage induction by gamma-irradiation, as an indication of intact HR function (Figure 1E–1F, Table 1). The results from cell lines representing each of our categories for resistant [OV1369(R2)], intermediate [TOV2295(R)] and sensitive (OV4453) response to Olaparib are shown in Figure 1E. Both OV1369(R2) and TOV2295(R) exhibited RAD51 foci with no radiation and the number of foci increased after radiation, indicating a functional HR response. In contrast, OV4453 displayed no foci, with or without irradiation, demonstrating a non-functional HR response. RAD51 foci response for all cell lines was ordered according to Olaparib sensitivity to infer any relationship between PARPi response (sensitive, intermediate, resistant) and HR functionality (Figure 1F). To ensure that a non-functional HR response was not due to lack of DNA double-strand break (DSB) induction after gamma-irradiation, γ-H2AX foci were evaluated as this protein is the first step in recruiting and localizing DSB repair proteins and can be used as a biomarker for this type of damage [35]. Our results (Figure S2) show that more than three-fold increase in γ-H2AX foci was observed in all cell lines studied after gamma-irradiation, indicating effective DNA damage induction. Overall, our findings indicated that increasing the levels of Olaparib resistance were correlated with greater HR function such that resistant cells were more likely to have functional HR than intermediate and sensitive cells. As expected, both *BRCA1* and *BRCA2* mutated cell lines (OV4485 and OV4453, respectively) had impaired HR function. Therefore, our correlation between HR functionality and PARPi responsiveness supports the current literature [19, 27, 32–34]. However, not all cell lines followed this correlation, as there was no significant difference between the Olaparib sensitive and intermediate cell lines based on RAD51 foci number (Figure S1C). For example, some intermediate cell lines showed the same level of HR functionality as resistant cell lines, while some intermediate cell lines showed little to no HR functionality [such as OV4485, OV3133(R) and TOV3133G]. We also noted that although our control *BRCA1* mutated cell line had deficient HR function, this cell line did not demonstrate the highest sensitivity to Olaparib. Since HR works with sister chromatids, we verified whether the RAD51 foci staining was indeed localized to cells in the late S/G2 mitotic phases by co-staining with antibody against geminin as previously described [36]. Geminin is a nuclear protein that is absent during G1 phase and that accumulates through S, G2 and M phases of the cell cycle [37]. The number of cells with double positive RAD51 and geminin staining were counted before and after gamma-irradiation in six cell lines [two of each group, i.e. sensitive, OV1946, TOV3041G; intermediate, TOV2295(R), OV4485; and resistant, OV1369(R2), OV866(2)] (Figure S3). Our results show the exact same profile as that shown in Figure 1F, indicating that our evaluation of HR function by counting RAD51 foci is accurate.

In all, our observations demonstrate that HR deficiency is not the only predictor of sensitivity to PARPi, and indicates that other DNA repair pathways, regardless of association with HR, may also contribute to PARPi responsiveness.

### Olaparib sensitive cell lines have deregulated gene expression in multiple DNA repair pathways

**Figure 2:**
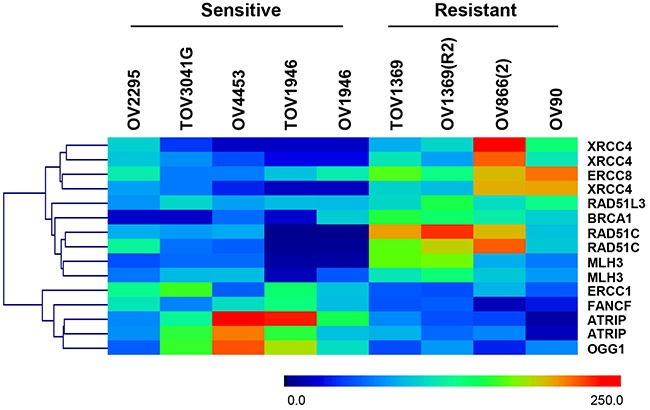
Heat map representation and gene clustering of differentially expressed DNA repair genes in sensitive and resistant HGS EOC cell lines Scale 0 to 250 represents normalized expression values of the Affymetrix gene expression array.

To establish whether other DNA repair pathways could play a role in Olaparib sensitivity of HGS EOC cell lines, we analyzed gene expression of DNA repair genes and pathways using a microarray approach. Two types of analyses were performed (see methods for details), one where each sensitive cell line was individually compared to all the resistant cell lines (Table 2), which takes into consideration the particularity of each cell line, and another where genes commonly deregulated in all sensitive cell lines were examined (Figure 2). For the first approach, we used the total gene expression dataset to identify genes that were up- or down-regulated by two-fold when comparing each sensitive cell line to all the resistant cell lines, and differentially expressed genes were annotated using the IPA program to identify significantly affected DNA repair pathways (*p* < 0.05). Our results show that each sensitive cell line had one or more DNA repair pathways that were differentially regulated when compared to the resistant cell lines (Table 2). Key genes of the MMR pathway such as *MLH1*, *MSH2* and *MSH6* were consistently down-regulated among the sensitive cell lines. However, less conclusive results were obtained for the non-homologous end joining (NHEJ) pathway since key genes of this pathway (*LIG4* and *DCLRE1C*) were both up- and down-regulated.

**Table 2:** Deregulated DNA repair pathways between Olaparib sensitive and resistant cell lines

Cell line	DNA repair ingenuity canonical pathways	p-value	Upregulated genes	Downregulated genes
OV2295	NHEJ	0.0035	DCLRE1C, LIG4	
OV4453	MMR	0.045	RPA1	EXO1
TOV1946	MMR	5.25e^-10^		MLH1, FEN1, RFC3, EXO1, POLD1, PCNA, RFC1, RFC5, MSH6, RFC4, MSH2
	HR	4.68e^-07^		BRCA1, POLA1, RAD50, ATM, RPA1, LIG1, RAD51, ATRX, MRE11A, NBN
	NHEJ	6.92e^-06^		PARP1, RAD50, XRCC5, ATM, XRCC4, DCLRE1C, MRE11A, LIG4, NBN
TOV3041G	HR	8.51e^-06^	LIG1, RAD51	BRCA1, RAD50, ATRX, MRE11A, NBN
	NHEJ	1.15e^-04^		RAD50, XRCC4, DCLRE1C, MRE11A, LIG4, NBN
	MMR	0.015		RFC3, MSH6, MSH2
OV1946	MMR	6.61e^-10^		MLH1, FEN1, RFC3, EXO1, MSH6, RFC5, POLD1, RFC4, PCNA, MSH2
	HR	0.0074		RPA1, RAD51, ATRX, MRE11A, NBN
	NHEJ	0.0074		PARP1, XRCC4, MRE11A, LIG4, NBN
	NER	0.044		POLR2B, RPA2, POLR2J, POLR2C, POLR2I, POLR2K

We next examined whether distinct DNA repair genes were commonly deregulated in all the sensitive cell lines. In this approach, mean expression values of specific DNA repair genes (155 genes, 240 probes) (see Supplementary Table S1), selected based on previously published data [26, 38], were analyzed. Heat map visualization and clustering of genes with significant differences in expression (*p* < 0.05, Mann Whitney) between the two groups are shown in Figure 2. Our results showed that four genes (*ERCC1*, *FANCF*, *ATRIP*, and *OGG1*) were significantly up-regulated whereas six genes (*XRCC4*, *ERCC8*, *RAD51D*, *BRCA1*, *RAD51C*, and *MLH3*), were significantly down-regulated in the sensitive cell lines compared to resistant cell lines. As per our first approach, we observed de-regulation of multiple DNA repair pathways (HR, NER, MMR and NHEJ) in Olaparib sensitive cell lines when compared to resistant cells.

### Increased Olaparib sensitivity upon down-regulation of MMR and NER genes in HGS cells

Although the base excision repair (BER) and NHEJ pathways have been implicated in PARPi response [39, 40], our results did not reveal consistent gene alteration in these pathways when comparing sensitive to resistant HGS EOC cell lines. On the other hand, the relationship between PARPi and the MMR or NER pathways is still not clearly defined or well understood [41–44]. Therefore we explored these pathways as possible mechanisms, other than HR, that may contribute to the Olaparib sensitivity of HGS EOC cell lines. From our list of differentially expressed genes we focused on *MLH1* and *MLH3* from the MMR pathway and *ERCC8* from the NER pathway. *MRE11A*, shown to be an important gene in PARPi sensitivity [26, 43], was selected to represent the HR pathway to examine any association between the HR pathway and the MMR and NER pathways in Olaparib sensitivity.

**Figure 3:**
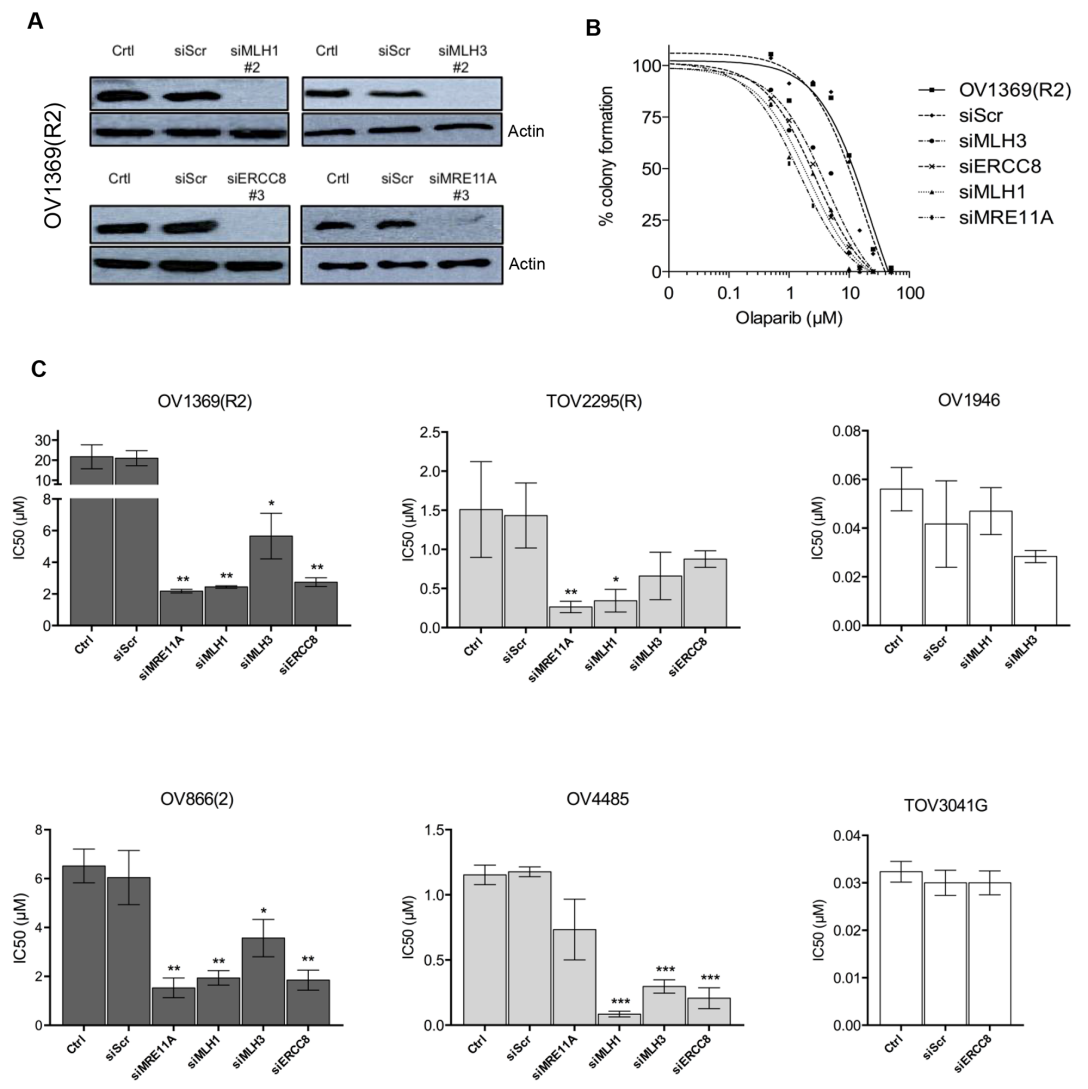
Validation by siRNA silencing of candidate genes contributing to Olaparib sensitivity **A**. Efficacy of siRNAs against *MLH1*, *MLH3*, *ERCC8* and *MRE11A* in OV1369(R2) was verified by Western blot. Control = non-treated cells, siScr = negative siRNA control using a scrambled sequence. **B**. Increased Olaparib sensitivity of OV1369(R2) with siRNAs against *MRE11A*, *MLH1*, *MLH3* and *ERCC8*, assayed by clonogenic assay. **C**. Verification of *MLH1*, *MLH3*, *ERCC8* and *MRE11* as candidate genes by siRNA knockdown in resistant [OV1369(R2) and OV866(2); dark grey bars], intermediate [TOV2295(R) and OV4485; light grey bars] and control sensitive (OV1946 and TOV3041G; white bars) cell lines. Bars represent average ± SEM of IC_50_ values obtained by clonogenic assay. * denotes *p* < 0.05, ** *p* < 0.01, *** *p* < 0.001.

Having selected target genes that are potentially associated with PARPi sensitivity, we performed validation experiments using siRNA knockdowns. Western blot analysis demonstrated that siRNAs against *MRE11A*, *MLH1*, *MLH3* and *ERCC8* efficiently down-regulated their protein targets in the resistant OV1369(R2) cell line (Figure 3A and Figure S4). Olaparib dose-response curves of this cell line were altered with the knockdown of all four genes (Figure 3B), indicating a decreased IC_50_ and increased sensitivity. Having validated the efficacy of our siRNAs, additional clonogenic assay experiments were performed in two resistant [OV1369(R2) and OV866(2)] and in two intermediate [TOV2295(R) and OV4485] cell lines to further verify whether these siRNAs could increase sensitivity to Olaparib. Figure 3C shows a significant decrease in the Olaparib IC_50_ of both resistant cell lines upon downregulation of each of the four selected genes. More precisely, down-regulation of *MLH1* (MMR pathway), *ERCC8* (NER pathway) or *MRE11A* (HR pathway) induced an approximate 10-fold decrease in the Olaparib IC_50_ (Table 3), which were closer to IC_50_ values observed for intermediate cell lines that were not subjected to siRNA knockdown. *MRE11A* knockdowns shifted the dose-response curves of the resistant cell lines closer to the intermediate but not to the sensitive group, which supports our initial hypothesis that defects in HR alone is insufficient for high sensitivity to PARPi. Although IC_50_ values of resistant cell lines were significantly decreased by all siRNA knockdowns, *MLH3* was the least effective of the four selected gene targets (Figure 3C, Table 3). The lack of efficacy with *MLH3* siRNA may be in part due to MLH3 having a minor role in the MMR pathway [45]. To ensure that our results were not due to off target effects of the chosen siRNAs, clonogenic assays were performed in these two resistant cell lines using a different siRNA sequence for each gene. Figure S5 shows the same inhibition profile as that shown in Figure 3C, indicating the specificity of our siRNAs.

**Table 3:** Quantification of Olaparib sensitivities (IC_50_) in EOC cell lines upon gene downregulation of the selected genes by siRNA

siRNA		Olaparib IC_50 _(µM)								
		Control	Scr	MRE11A	MLH1	MLH3	ERCC8	MRE11A /MLH1	MRE11A /ERCC8	MLH1 /ERCC8
Resistant	OV1369(R2)	21.71 ±10.33	21.03 ±6.44	2.18 ±0.19	2.44 ±0.13	5.66 ±2.50	2.74 ±0.48	0.03 ±0.02	0.08 ±0.03	0.80 ±0.06
	OV866(2)	8.11 ±1.27	6.04 ±1.10	1.53 ±0.40	1.94 ±0.29	3.57 ±0.76	1.85 ±0.41	0.01 ±0.005	0.05 ±0.02	1.10 ±0.70
Intermediate	TOV2295(R)	1.52 ±1.14	1.43 ±0.42	0.26 ±0.07	0.34 ±0.15	0.66 ±0.30	0.88 ±0.11	0.11 ±0.08	0.09 ±0.06	0.20 ±0.07
	OV4485	0.90 ±0.58	1.18 ±0.07	0.73 ±0.40	0.08 ±0.04	0.30 ±0.05	0.21 ±0.08	0.06 ±0.01	0.06 ±0.004	0.08 ±0.02
Sensitive	OV1946	0.07 ±0.05	0.04 ±0.02	N/A	0.05 ±0.01	0.03 ±0.003	N/A	N/A	N/A	N/A
	TOV3041G	0.02 ±0.01	0.03 ±0.005	N/A	N/A	N/A	0.03 ±0.004	N/A	N/A	N/A

The two intermediate cell lines [TOV2295(R), OV4485] also demonstrated a decrease in Olaparib IC_50_ when target genes were down-regulated, with values shifting from the intermediate to the sensitive range (Figure 3C, Table 3). However, results were less striking than those obtained with the resistant cell lines, as not all gene knockdowns induced significant effects. The *BRCA1* mutated cell line, OV4485, is of special interest as these cells are compromised in HR function (Figure 1F). As expected, siMRE11A was unable to induce a significant decrease in Olaparib sensitivity since the HR pathway was already non-functional. However siRNAs against *MLH1*, *MLH3*, and *ERCC8* induced substantial decreases in the IC_50_ values (Figure 3C, Table 3). These extreme drops in IC_50_ values could indicate that deficiency of two DNA repair pathways may be necessary to achieve greater Olaparib sensitivity. In TOV2295(R) cells, siRNA against both *MRE11A* and *MLH1*, but not *MLH3* and *ERCC8*, produced a significant decrease in Olaparib IC_50_. The insignificant effect of *ERCC8* down-regulation in this cell line was not expected and we speculate that the NER pathway could be compromised. A recent study has described that 8% of HGS EOC exhibited mutations or homozygous deletions of NER genes [46]. Further investigation of the NER function in this cell line is warranted. Similar to the resistant cell lines, *MLH3* down-regulation was less effective than *MLH1* in increasing Olaparib sensitivity in both intermediate cell lines.

To further validate the siRNAs and their targets, siMLH1 and siMLH3 were tested in OV1946 a sensitive cell line that had lower expression for these two proteins based on gene expression microarray analyses relative to resistant cell lines (Table 2 and Figure 2). As expected, there was no change to PARPi sensitivity (Figure 3C). A similar result was observed with siERCC8 in TOV3041G cells (Figure 3C), which demonstrated lower ERCC8 expression by gene expression microarray analyses relative to resistant cell lines (Figure 2).

In order to demonstrate that silencing of MMR and NER genes do not affect the HR function, RAD51 foci were evaluated after *MLH1*, *MLH3* and *ERCC8* knockdown in HR proficient OV866(2) and OV1369(R2). As expected, knocking down these genes did not alter RAD51 foci number in total or geminin-positive cells (Figure S6). On the other hand, *MRE11A* siRNA efficiently impaired HR function in both cell lines studied (Supplementary Figure S6).

### Increased sensitivity to Olaparib involves defects in HR plus another DNA repair pathway

**Figure 4:**
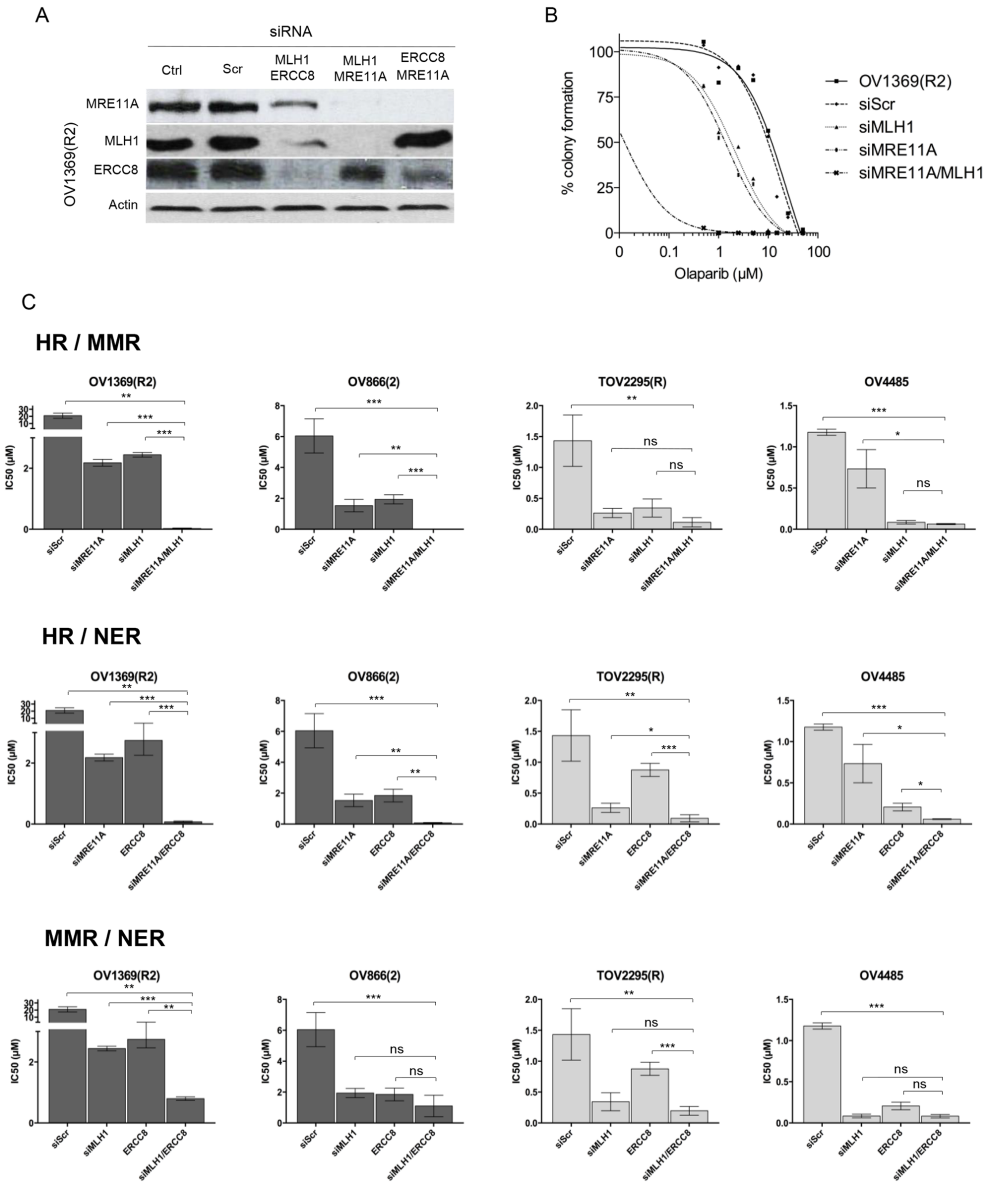
Olaparib sensitivity is enhanced by non-functional HR combined with defects in another DNA repair pathway **A**. siRNAs efficacy against MLH1, ERCC8, and MRE11A in combination, in OV1369(R2) cells was verified by Western blots. **B**. Olaparib sensitivity curves of OV1369(R2) representing non-treated cells, control siRNA (siScr), and single and double siRNAs against *MRE11A* and *MLH1* together. **C**. IC_50_ representations of single and double siRNAs in resistant (dark grey bars), and intermediate (light grey bars) cells. Bars represent average ± SEM of IC_50_ values obtained by clonogenic assay. * denotes p < 0.05, ** p < 0.01, *** p < 0.001, ns = non-significant.

Our initial siRNA results indicate that more than one defective DNA repair pathway is necessary for cells to become highly sensitive to PARPi. This would explain the observation that some patients with *BRCA* mutations respond to PARPi while others do not. To this end we used siRNAs in combination to investigate their effect on the Olaparib response. Due to the less pronounced effects of *MLH3* down-regulation on Olaparib sensitivity, siRNA against this gene was not used for double knockdown assays. Therefore, siRNAs against *MRE11A*, *MLH1*, and *ERCC8* were used in combination. Efficacy of the siRNA combination was verified by Western blot and compared to each single siRNA treatment (Figures 3A and 4A). All siRNA combinations showed efficient knockdowns of their targets except for siERCC8 combined with siMRE11A, which was not as effective as siERCC8 alone for *ERCC8* knockdown. Nevertheless, the double knockdown still induced diminished protein expression compared to control scramble siRNA treated cells. When the representative resistant cell line OV1369(R2) was assayed for Olaparib sensitivity, single siRNA treatment against *MRE11A* or *MLH1* resulted in an increase in sensitivity, while a double knockdown of *MRE11A* and *MLH1* displayed an even greater enhancement when compared to the parental cell line (Figure 4B).

Two resistant and two intermediate cell lines were then used to test three different combinations of siRNAs (Figure 4C). The first combination was used to impair the HR and MMR pathways (siMRE11A/siMLH1) in resistant cell lines. As previously shown, each siRNA alone could modify resistant cells to respond as intermediate cells when exposed to Olaparib. However, IC_50_ values fell into the range of sensitive cells when the two siRNAs were used in combination (Figure 4C and Table 3). A similar effect was observed when both HR and NER pathways were impaired (siMRE11A/siERCC8). Interestingly, this was not the case for the double impairment of the MMR and NER pathways (siMLH1/siERCC8). In the resistant cell line OV1369(R2), a double knockdown further decreased the IC_50_ from levels achieved by a single knockdown, but still fell in the range of the intermediate IC_50_ values (Table 3). In the other resistant cell line OV866(2), the double knockdown did not drop the IC_50_ significantly from values that were obtained with single knockdowns (Figure 4C and Table 3).

When HR and MMR were targeted (siMRE11A/siMLH1) in intermediate cells, TOV2295(R) did not display a significant change in sensitivity between single and double knockdowns (Figure 4C and Table 3). The same was observed for OV4485 when comparing double knockdown and siMLH1. However, siMLH1 alone or in combination with siMRE11A significantly enhanced Olaparib sensitivity compared to single *MRE11A* knockdown. As already stated, siMRE11A did not have a major effect, most likely because this cell line is a *BRCA1* mutant and already has non-functional HR. When both HR and NER were impaired (siMRE11A/siERCC8) the double knockdown was more effective than each single knockdown in both the TOV2295(R) and OV4485 cells (Figure 4C and Table 3). In the combined impairment of MMR and NER (siMLH1/siERCC8), TOV2295(R) cells did not have a strong reaction to siERCC8 alone, while both siMLH1 and siMLH1/siERCC8 showed increased sensitivity that were of similar levels reflecting a possible inherent defect in NER in this cell line. OV4485 cells impaired in MMR and NER pathways showed equally strong response to Olaparib in reactions affected by both single and double knockdowns (Figure 4C and Table 3), indicating no enhanced sensitivity due to the impairment of both pathways, as observed in the resistant OV866(2) cell line.

These findings suggest that defective HR is vitally important for PARPi sensitivity, but insufficient on its own to render a cell fully sensitive to PARPi and that a second pathway must be deregulated (either MMR or NER) to achieve the highest sensitivity observed *in vitro*. However, based on results with OV4485, once HR and one other pathway are compromised, there is no added benefit to a third pathway being compromised.

### Expression of targets in clinical samples

**Figure 5:**
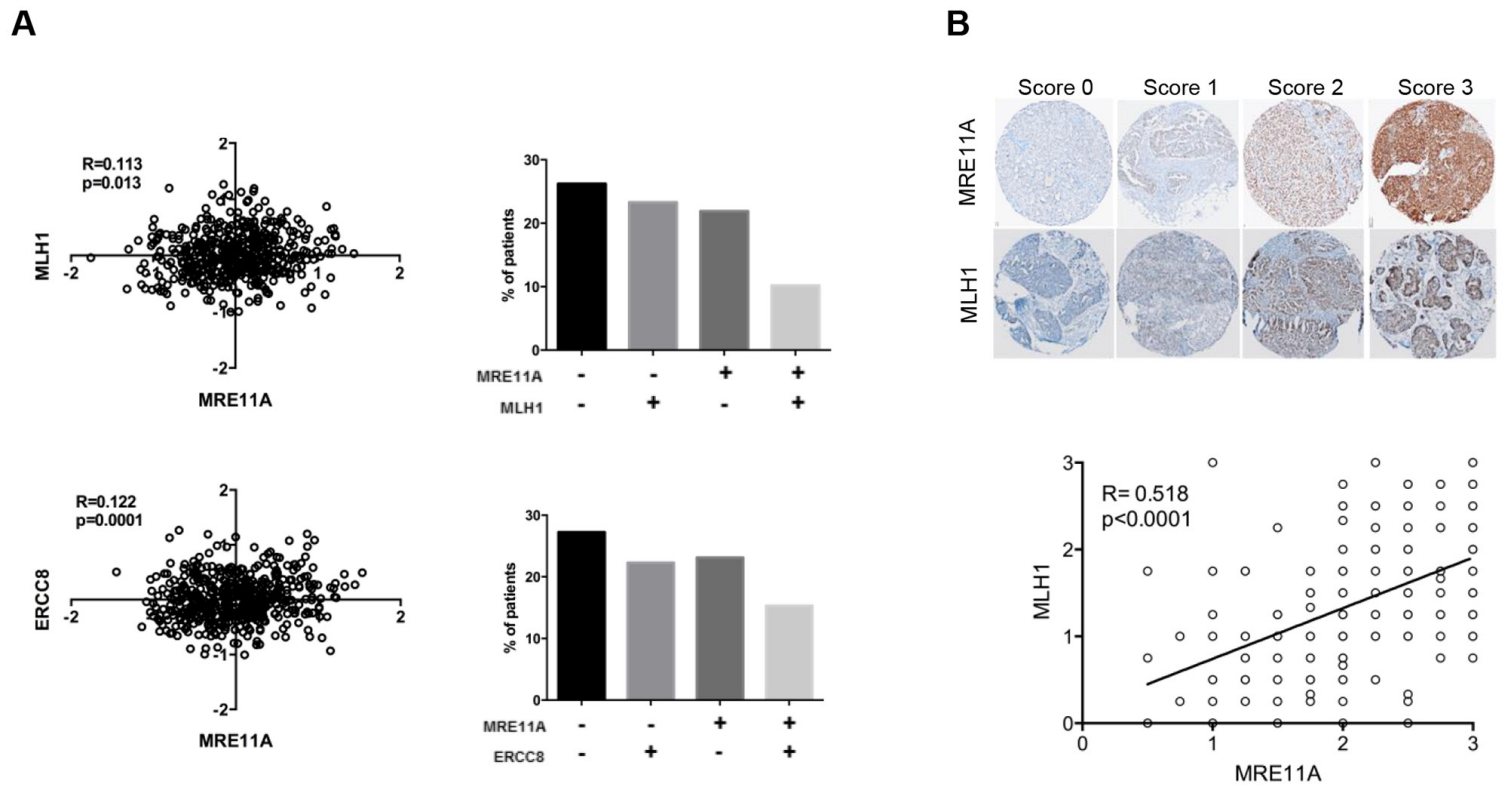
Correlations between expression levels of DNA repair genes in clinical samples **A**. Correlations between gene expression levels of *MRE11A/MLH1* and *MRE11A/ERCC8* using the TCGA dataset of HGS tumors. Bars represent percent of patients in each category: double negative, single positive or double positive biomarkers staining. **B**. Upper panel shows representative images of IHC scores (0, 1, 2, 3) for MLH1 or MRE11A staining. Bottom panel shows correlation between expression levels of MLH1 and MRE11A proteins in a TMA containing patients’ samples of HGS EOC.

After observing that HR and another DNA pathway must be defective for optimal PARPi sensitivity, we sought to determine the co-occurrence of deficiencies in these pathways in clinical EOC specimens by examining the co-expression of MHL1 and MRE11A, or ERCC8 and MRE11A. The correlation analyses were performed on the gene expression microarray data from more than 400 HGS EOC patients available through The Cancer Genome Atlas Research (TCGA) Network [18] (Figure 5A). Our results showed significant Pearson correlations between *MRE11A* and *MLH1* expression (p=0.013, R=0.113) and *MRE11A* and *ERCC8* expression (p=0.0001, R=0.122), indicating that defects in HR and MMR/NER pathways could co-occur in clinical specimens. Approximately 25% of the specimens exhibited concomitant low expression levels of these biomarkers pairs (Figure 5A), and according to our *in vitro* results (Figure 4C), patients with these cancers would potentially have a better response to Olaparib. At the protein level, we analyzed the expression of these by immunohistochemistry (IHC) using a tissue microarray (TMA) containing 213 HGS ovarian tumors. Specificity of antibodies against MLH1 and MRE11A are shown in Figure S7. However, due to poor antibody specificity, ERCC8 was not assayed in the TMA. Our results showed a strong Pearson correlation between the expression of MRE11A and MLH1 proteins (*p*<0.0001, R=0.518) (Figure 5B), and we found 17.5% patients having low levels (scores≤1.5) of both of these proteins. Our findings suggest that the investigation of these genes as biomarkers in clinical specimens could help identify HGS EOC patients most likely to respond to PARPi treatment.

## DISCUSSION

PARP1 exerts significant effects on several biological functions that are critical for cell growth and survival [7–9]. In the context of DNA damage, PARP1 binds damaged DNA and undergoes a conformational change resulting in its activation. Once activated, PARP1 synthesizes PAR chains that covalently bind a variety of chromatin-associated proteins, although PARP1 itself is the major acceptor of the PAR chains. The resulting PARylation not only alters the function of covalently bound proteins but can also stimulate the recruitment of a wide variety of other DNA nuclear proteins. PAR levels reflect DNA damage that is present, but once DNA repair ensues, PAR is rapidly degraded by PAR glycohydrolases. Through PAR synthesis, PARP1 contributes to a number of DNA repair pathways [7–9]. PARP1 plays a role in the BER, is involved in the alternative end joining repair pathway, recruits MRE11A and NSB1 to initiate HR, PARylates BRCA1 that further contributes to the HR pathway, and prevents NHEJ activation. Therefore, in the context of PARP1 inhibition, it is clear that response to PARPi extends beyond the BRCA1/2 mutation- and HR-status.

**Figure 6:**
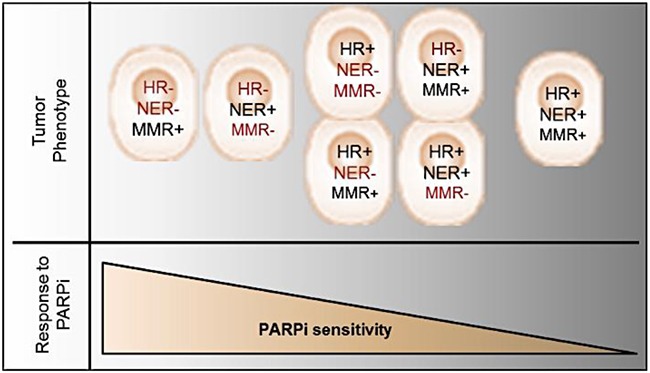
Schematic model representing tumor phenotype and corresponding PARPi response The synthetic lethality resulting in PARPi sensitivity is presented if HR deregulation is concomitant with another DNA repair pathway deficiency.

Based on present research findings, we propose a new model to account for the spectrum of PARPi responsiveness that is dependent on the types of DNA pathways affected in EOC (Figure 6). Tumors with phenotypes that include either defective HR and NER, or defective HR and MMR pathways, should respond with the highest sensitivity to PARPi, in keeping with the concept of synthetic lethality. Tumor phenotypes with only one defective DNA repair pathway may show limited PARPi responsiveness, whereas patients with tumor phenotypes with functionally intact DNA repair pathways would not respond to PARPi treatment. Indeed, the ARPE19 normal epithelial retina cell line demonstrates an IC_50_ comparable to EOC Olaparib resistant cell lines (data not shown). Similar to the HR pathway, the MMR and NER pathways are involved in the repair of DSB [47], such that DSB repair is reduced when MMR or NER are deficient [44, 47, 48]. Also, it has been demonstrated that PARP1 acts directly on the activity of the NER and MMR [49, 50]. However, only few reports have described the role of the MMR and NER pathways in the response to PARPi [41–44, 51]. Our work has demonstrated that down-regulation of genes involved in the MMR or NER pathways increased Olaparib sensitivity of HGS EOC cell lines, even in *BRCA* mutated cell lines (Figure 3). In addition, our results suggest that the MMR and the NER pathways might repair DSB when HR is defective in tumor cells, and may explain why some patients harboring a *BRCA* mutation do not respond to PARPi treatment. Alternatively, tumors cells having defects in HR and in either MMR or NER pathways are more sensitive. However, we did not observe a significant increase in Olaparib sensitivity when MMR and NER were both deficient in cells with intact HR, indicating that DSB are mainly repaired by the HR pathway when either one of these pathways are defective.

Although other studies have correlated NER deficiency with PARPi response [42, 51], this is the first report showing *ERCC8* as a potential target for PARPi sensitivity. NER deficiency has also been implicated in the response to platinum-based therapy in EOC patients [46, 52], which is consistent with the observation that platinum-sensitive patients also respond to PARPi treatment [13, 29]. Although a recent report showed that mutations in the NER genes *ERCC6* and *ERCC4* did not increase PARPi sensitivity to Rucaparib [46], our results showed that under-expression of *ERCC8* significantly increased Olaparib sensitivity in HGS cells that were either HR-deficient or -proficient. In the case of *MLH1*, reports have shown that MMR deficiency confers platinum resistance [53], whereas our results showed that down-regulation of *MLH1* resulted in a significant increase in Olaparib sensitivity. Therefore, our findings suggest that platinum-resistant patients with low *MLH1* expression may benefit from PARPi treatment. Indeed, the TOV1946 cell line exhibiting very low levels of *MLH1* (Figure 2) is very sensitive to Olaparib (IC_50_=0.02 μM), and our previous work has shown that this cell line is not very sensitive to carboplatin (IC_50_=4 μM) [23].

Analysis of gene (TCGA dataset) and protein (TMA) expression in HGS EOC patient samples showed a significant correlation between *MRE11A* (HR pathway) and *MLH1* (MMR pathway) or *ERCC8* (NER pathway) (Figure 5). Therefore, it may be possible to identify patients with tumors containing low levels of *MRE11A* and *MLH1* or *ERCC8*, and distinguish them as patients who may respond better to Olaparib. In the case of patients with *BRCA* mutations, low levels of either *MLH1* or *ERCC8* may predict a better response to Olaparib treatment, as demonstrated by our results with siRNA against either *MLH1* or *ERCC8* in a *BRCA1* mutated cell line (OV4485) (Figure 3). These findings could impact the clinical management of HGS EOC patients such that patients that have these molecular tumor characteristics, could be treated preferentially with Olaparib, and possibly at lower doses. Phase II clinical trials of Olaparib have been performed with 100, 200 or 400 mg twice daily where a 100 mg dose resulted in a response rate of 12% and a 200 or 400 mg dose resulted in a response of around 30-40% [12, 29, 54]. However, only the dose of 400 mg twice daily was included in a clinical trial of Olaparib as maintenance therapy in a large cohort with platinum-sensitive *BRCA* mutated HGS EOC; although improved survival was observed for the Olaparib group, severe side effects were reported [13, 14]. Therefore, stratifying HGS patients may help to increase the quality of life for some patients by decreasing drug dosage. In our model, stratification of patients with “BRCAness” plus defective MMR or NER may identify those who could be treated with the lowest doses.

A microarray study characterizing Olaparib sensitivity in breast cancer cell lines have identified six DNA repair genes that were significantly down-regulated (*MRE11A*, *NBS1*, *TNKS*, *TNKS2*, *XPA*, *XRCC5*) and two that were significantly up-regulated (*CHEK2*, *MK2*) in sensitive cell lines relative to resistant cell lines [26]. *MRE11A* is the only gene in common between this study and our work (Table 2). However, the authors also identified a gene from the NER pathway (*XPA* gene) that was significantly down-regulated in sensitive cells, indicating that deregulation of this pathway affects PARPi response in both cancer types. Therefore, results from this study are consistent with our proposed model since they reported defects in HR and NER pathways in Olaparib-sensitive breast cancer cell lines. In contrast, MMR genes did not appear to be deregulated in breast cancer cells, highlighting the different roles of this pathway in breast and ovarian cancers. Although other potential biomarker genes in DNA repair have been described in association with Olaparib sensitivity [38, 55], our work is particularly noteworthy for the identification of defects in several DNA repair pathways that demonstrated a cumulative effect in our cell line models resulting in acute Olaparib sensitivity.

Furthermore, our findings provide evidence that proficiency in NER or MMR may account for mechanisms of PARPi resistance, other than mutation reversal or p-glycoprotein up-regulation [55], to explain why BRCA mutated EOC patients do not respond favorably to Olaparib.

## MATERIALS AND METHODS

### Cell lines and cell culture

The 18 human HGS EOC cell lines used (OV90, OV866(2), TOV1369, OV1369(R2), TOV1946, OV1946, TOV2223G, OV2295, OV2295(R2), TOV2295(R), TOV2978G, TOV3041G, TOV3291G, TOV3133G, TOV3133D, OV3133(R), OV4453, OV4485) were derived in our laboratory from patients’ tumors (TOV) or ascites (OV) [22–25]. All cell lines were maintained in a low oxygen condition of 7% O_2_ and 5% CO_2_ and grown in complete OSE medium, which contains OSE medium (Wisent, Montreal, QC) with 10% FBS (Wisent), 0.5 μg/mL amphotericin B (Wisent) and 50 μg/mL gentamicin (Life Technologies Inc., Burlington, ON).

### Antibodies

The following antibodies were used: beta-actin (AC-15) (ab6276; Abcam Inc., Toronto, ON, Canada); MLH1 (4C9C7) (3515; Cell Signaling Technology Inc., Danvers, MA); MLH3 (NBP1-00106; Novus Biologicals, Oakville, ON); ERCC8 (ABIN486848; Antibodies-online, Atlanta, GA); PAR (4335-MC-100; Trevigen®, Gaithersburg MD); PARP1 (H-250)(SC-7150, Santa Cruz Biotechnology Inc., Dallas, TX); MRE11A (4895, Cell Signaling Technology Inc.); RAD51 (14B4) (ab213; Abcam Inc.); phospho-histone γ-H2AX (Ser139) (JBW301) (05-636, Millipore, Temecula, CA); and Geminin (10802-1-AP, Proteintech, Rosemont, IL).

### Western blot

Thirty micrograms of total protein extracts were separated on 10% SDS-polyacrylamide gels and transferred onto nitrocellulose membrane. Membranes were blocked with 5% skim milk in PBS-Tween for 1 hour and probed with primary antibodies (diluted at 1:1000 for MLH1 or PARP1, 1:2000 for MLH3, 1:3000 for ERCC8 and 1:1000 for MRE11A) overnight at 4°C. After the addition of HRP-conjugated secondary antibodies, proteins were detected using enhanced chemiluminescence (ECL). Loading control for the samples was determined with anti- ß-actin antibody (1:10000).

### Analysis of PAR levels

The analysis of PAR levels was performed as previously described [56]. Briefly, cells were seeded in 6-well plates, and incubated for 24 hours before they were treated with 1mM H_2_O_2_ for 20 minutes. Then, cells were treated with 20µM or 40µM Olaparib for 24 hours, harvested and lysed to obtain protein extracts that were analyzed by Western blot using an anti-PAR antibody (1:1500).

### Cell cycle flow cytometry analysis

Cells were seeded in 6-well plates and grown to 70% confluence. Media was then removed and replaced with complete OSE media containing 40µM Olaparib. Cells were harvested 24 hours after, fixed in 70% ethanol and incubated (30 min, RT) with 100µg/ml RNAse A and 25µg/ml Propidium Iodide for cell cycle analysis. A maximum of 10,0000 events were counted per condition using the Fortessa flow cytometer (BD Biosciences, Mississauga, ON).

### Clonogenic survival assay to measure drug sensitivity

Olaparib (Selleckchem, Houston, TX) sensitivity of the cell lines was assessed using a concentration range of 0–50 µM on clonogenic assays as previously described [22, 23]. Colonies were counted under a stereo microscope and reported as percent of control. IC_50_ values were determined using Graph Pad Prism 5 software (GraphPad Software Inc., San Diego, CA). Each experiment was performed in triplicate and repeated three times. Cells were categorized as either sensitive, intermediate, or resistant to PARPi based on groupings of IC_50_.

### Analysis of nuclear RAD51 and γ-H2AX foci

Cells were seeded onto coverslips in 12-well plates, grown until 80% confluence, and gamma-irradiated at 8Gy for 2 hours for RAD51 or 2Gy for 1 hour for γ-H2AX foci analyses. These conditions were established to count an appropriate number of foci during the peak of recruitment of these proteins at the damaged DNA site after gamma-irradiation [57, 58]. Cells were then washed in ice-cold phosphate buffered saline (PBS), fixed in 4% paraformaldehyde and permeabilized with 0.25% Triton X-100 (Sigma–Aldrich Inc.). After blocking (5% BSA and 4% FBS in PBS), coverslips were incubated with the anti–RAD51 (1:500) or the anti-γ-H2AX (1:1500) primary antibodies, and subsequently incubated with Alexa Fluor 488 (1:500) or Cy-5 (1:800) secondary antibodies (Life Technologies Inc.) for RAD51 or γ-H2AX staining, respectively. Coverslips were mounted onto slides using Prolong^®^ Gold anti-fade reagent with DAPI (Life Technologies Inc.). Images were obtained using a Zeiss microscope (Zeiss observer Z1, Carl Zeiss, Jena, Germany). Automated analysis software from Zeiss (AxioVision™, Carl Zeiss) was used for foci counting. The average number of foci per nucleus was expressed as a percentage of the non-irradiated controls. Fold change was calculated as a ratio between percentages of RAD51 foci in treated over control non-treated cells. RAD51 foci were quantified in roughly 400 nuclei from three different fields of each coverslip. Reliability, reproducibility, and validity of our data were confirmed by repeated tests across different fields. For RAD51-geminin co-staining [36], coverslips were incubated with RAD51 (1:500) and Geminin (1:1000) primary antibodies and subsequently incubated with Cy-5 and Alexa Fluor 488 secondary antibodies, respectively.

### RNA preparation and gene expression microarray analyses

Four of the cell lines used in this study (OV90, TOV1946, OV1946, TOV2223G) had been previously subjected to gene microarray analyses using the Affymetrix Human Genome U133A [59, 60]. For the remaining 14 cell lines, RNA was extracted and purified as previously described [59, 60] and microarray experiments were performed at the McGill University and Genome Quebec Innovation Centre (genomequebec.mcgill.ca) using HG-U133 Plus 2.0 GeneChip arrays (Affymetrix®, Santa Clara, CA). Gene expression levels were calculated for each probe set from the scanned image by Affymetrix® GeneChip (MAS5) and prepared as previously described [61]. As reproducibility of expression values is highly variable at low values of expression [62], all normalized values below five were reassigned a threshold value of five based on the mean expression value of the lowest reliability scores. Probe sets having these threshold values in all the samples were not used for further the analysis. Further normalization across probe sets was performed in order to combine datasets from the previously analyzed HG-U133A (four cell lines) and the HG-U133 Plus 2.0 (14 cell lines). Altogether, there were more than 22,000 probe sets in common, covering 18,400 transcripts and variants, including 14,500 well-characterized human genes.

Microarray data were used to identify genes that were two fold up- or two fold down-regulated in expression for each sensitive cell line (OV2295, TOV3041G, OV4453, TOV1946 and OV1946) when compared to all resistant [OV90, OV886(2), TOV1369 and OV1369(R2)] cell lines. A list of genes was obtained for each sensitive cell line, following the strict criteria that genes must be differentially regulated when compared to all the other cell lines within the resistant group. Gene lists were uploaded onto the Ingenuity Pathway Analysis program (Qiagen Inc.) to obtain a list of canonical pathways affected in each cell line. Only significantly (*p*<0.05) affected DNA repair pathways were taken into account.

In another approach, probe sets of DNA repair genes were selected and mean expression values were analyzed to identify common differentially affected genes between Olaparib sensitive *versus* resistant cell lines using the non-parametric Mann-Whitney test. Significance was set at *p* value < 0.05. Visualization of gene expression values and gene clusters (heat maps) was performed using the MultiExperiment Viewer software (MeV_4_8_1 from TM4), a free, open-source tool for microarray analysis.

### Small interference RNA (siRNA) treatment

For each gene of interest, a collection of four different siRNAs (Dharmacon ON-TARGET plus siRNA, set of 4; Thermo Fisher Scientific Inc., Waltham, MA) (see Table S2 for sequences) was tested and the most effective were selected for further analysis (siRNA#2 for *MLH1* and *MLH3*; and siRNA#3 for *MRE11A* and *ERCC8*). To verify off target issues, a different siRNA for each gene was used in a subset of experiments (i.e., siRNA#4 for all genes). Scramble siRNA (Table S2) was used as control in all the experiments. Transfections were performed using the DharmaFECT® Transfection Reagents. Cells were transfected with 25 nM siRNA per well in a 6-well plate. For double siRNA treatment, 25 nM of each siRNA was added to the well at the same time. After 48 hours, cells were harvested and used for Western blot or clonogenic assays.

### Immunohistochemistry

In the present work we used a HGS EOC tissue microarray (TMA) containing 213 cases (two cores per patient), which has been previously described [63]. The TMA blocks were sectioned at 4μm thickness onto superfrost+ glass microscope slides (Fisher Scientific Limited, Nepean, ON, Canada), and stained using the BenchMark XT automated stainer (Ventana Medical System Inc., Tucson, AZ). Antigen retrieval for MLH1 and MRE11A was performed with Cell Conditioning Solution, CC1 (Ventana Medical System Inc.) for 30 minutes. Slides were incubated with anti-MLH1 (1:100) or anti-MRE11A (1:500) antibodies for 40 minutes, and developed with the iView DAB detection kit (Ventana Medical System Inc.). Hematoxylin and bluing reagent were used for counterstaining (Ventana Medical System Inc.). TMAs were observed by brightfield microscopy and digitally imaged (Aperio ScanScope, Vista, California, USA). Protein expression by IHC was scored according to the nuclear staining intensity in epithelial zones of the tumor cores. The staining intensity of DAB was defined as 0 (no staining), 1 (weak staining), 2 (moderate staining) and 3 (strong staining). All TMAs were analyzed in a blind study by two independent observers. Intra-class correlation (ICC) was greater than 75% for all assays. The average of all cores with cancer from the same patient was used for analysis. Graph Pad Prism 5 was used to perform Pearson correlation test (two-tailed) and significance was set at *p* < 0.05.

### TCGA dataset

The Unified Expression dataset of HGS EOC gene microarray from the TCGA was directly uploaded from The Cancer Genome Atlas (NCI/NIH) website (https://tcga-data.nci.nih.gov/docs/publications/ov_2011/) as a table format. This table contained normalized expression of 11864 genes from 489 HGS EOC samples as previously described [18]. Graph Pad Prism 5 was used to perform Pearson correlation test (two-tailed) and significance was set at *p* < 0.05.

## SUPPLEMENTARY MATERIALS FIGURES AND TABLES


