# Supplementary material for: Cumulative defects in DNA repair pathways drive the PARP inhibitor response in high-grade serous epithelial ovarian cancer cell lines
Source: Oncotarget. 2016 Jun 27;8(25):40152–68. doi: 10.18632/oncotarget.10308 (PMC5522225; doi:10.18632/oncotarget.10308)
Supplement: Supplementary file 1 [file oncotarget-08-40152-s001.pdf]

Cumulative defects in DNA repair pathways drive the PARP inhibitor response in high-grade serous epithelial ovarian cancer cell lines

SUPPLEMENTARY FIGURES AND TABLES

A. Student's t-test analysis (p value) – Olaparib IC_{50}

	OV1369(R2)	TOV1369	OV866(2)	OV90	TOV2223G	OV3133D	OV2295(R2)	TOV3291G	TOV2295(R)	OV4485	OV3133(R)	TOV3133G	TOV2978G	OV1946	TOV3041G	TOV1946	OV4453	OV2295
OV1369(R2)		1,2E-01	8,9E-02	7,8E-02	2,9E-02	3,7E-02	3,1E-02	2,9E-02	2,9E-02	2,6E-02	2,5E-02	2,5E-02	2,4E-02	2,3E-02	2,3E-02	2,3E-02	2,3E-02	2,3E-02
TOV1369			6,9E-01	4,7E-01	2,6E-02	5,3E-02	3,2E-02	2,4E-02	2,8E-02	1,9E-02	1,7E-02	1,7E-02	1,6E-02	1,3E-02	1,3E-02	1,3E-02	1,3E-02	1,3E-02
OV866(2)				5,1E-01	6,1E-04	4,2E-03	9,8E-04	2,5E-04	1,5E-03	3,1E-04	1,5E-04	2,0E-04	1,6E-04	1,1E-04	1,0E-04	1,0E-04	1,0E-04	1,0E-04
OV90					1,8E-02	5,5E-02	2,4E-02	1,5E-02	2,1E-02	1,1E-02	9,4E-03	9,1E-03	8,2E-03	6,6E-03	6,4E-03	6,4E-03	6,3E-03	6,3E-03
TOV2223G						1,6E-01	5,6E-01	9,0E-01	9,2E-01	2,5E-01	1,0E-01	9,7E-02	5,9E-02	2,0E-02	1,7E-02	1,7E-02	1,7E-02	1,7E-02
OV3133D							3,0E-01	1,1E-01	2,0E-01	5,3E-02	3,1E-02	3,0E-02	2,3E-02	1,3E-02	1,3E-02	1,3E-02	1,2E-02	1,2E-02
OV2295(R2)								4,0E-01	5,9E-01	1,3E-01	5,6E-02	5,6E-02	3,6E-02	1,5E-02	1,4E-02	1,4E-02	1,3E-02	1,3E-02
TOV3291G									9,7E-01	1,3E-01	1,2E-03	2,2E-02	5,0E-03	1,2E-04	9,0E-05	8,9E-05	8,6E-05	8,4E-05
TOV2295(R)										4,5E-01	3,0E-01	2,5E-01	1,9E-01	9,3E-02	8,5E-02	8,5E-02	8,4E-02	8,2E-02
OV4485											6,7E-01	4,8E-01	2,9E-01	6,8E-02	5,7E-02	5,7E-02	5,5E-02	5,4E-02
OV3133(R)												5,4E-01	1,6E-01	4,2E-05	8,6E-06	6,3E-06	5,7E-06	5,4E-06
TOV3133G													7,0E-01	1,2E-01	9,1E-02	9,1E-02	8,8E-02	8,4E-02
TOV2978G														9,8E-02	6,8E-02	6,8E-02	6,4E-02	6,0E-02
OV1946															1,2E-01	1,1E-01	7,2E-02	4,8E-02
TOV3041G																9,7E-01	4,5E-01	1,4E-01
TOV1946																	1,2E-01	1,1E-02
OV4453																		5,1E-05
OV2295																		

B. Student's t-test analysis (p value) – Olaparib IC_{50}

Comparison	Difference	q	*p* value	Significance level
sensitive vs intermediate	-1,514	4,215	P<0.01	**
sensitive vs resistant	-11,446	7,637	P<0.001	***
Intermediate vs resistant	-9,932	7,398	P<0.001	***

C. Student's t-test analysis (p value) – RAD51 foci

Comparison	Difference	q	*p* value	Significance level
sensitive vs intermediate	-26.733	1.189	P>0.05	ns
sensitive vs resistant	-200.15	7.399	P<0.001	***
Intermediate vs resistant	-173.42	4.157	P<0.05	*

Supplementary Figure S1: Statistical analysis for determining Olaparib sensitivity groups. A. Student's t-test analysis of IC_{50} values comparing a cell line with each of the other ones. Red fonts indicate the shift of significant difference ($p<0.05$) that was used to define the three groups of Olaparib sensitivity. **B.** Student's t-test comparison between the IC_{50} values of the three groups of sensitivity. **C.** Student's t-test comparison of the increase in RAD51 foci in the three groups of sensitivity. For both tables (B and C), * denotes $p < 0.05$, ** $p < 0.01$, *** $p < 0.001$ and ns = non-significant.

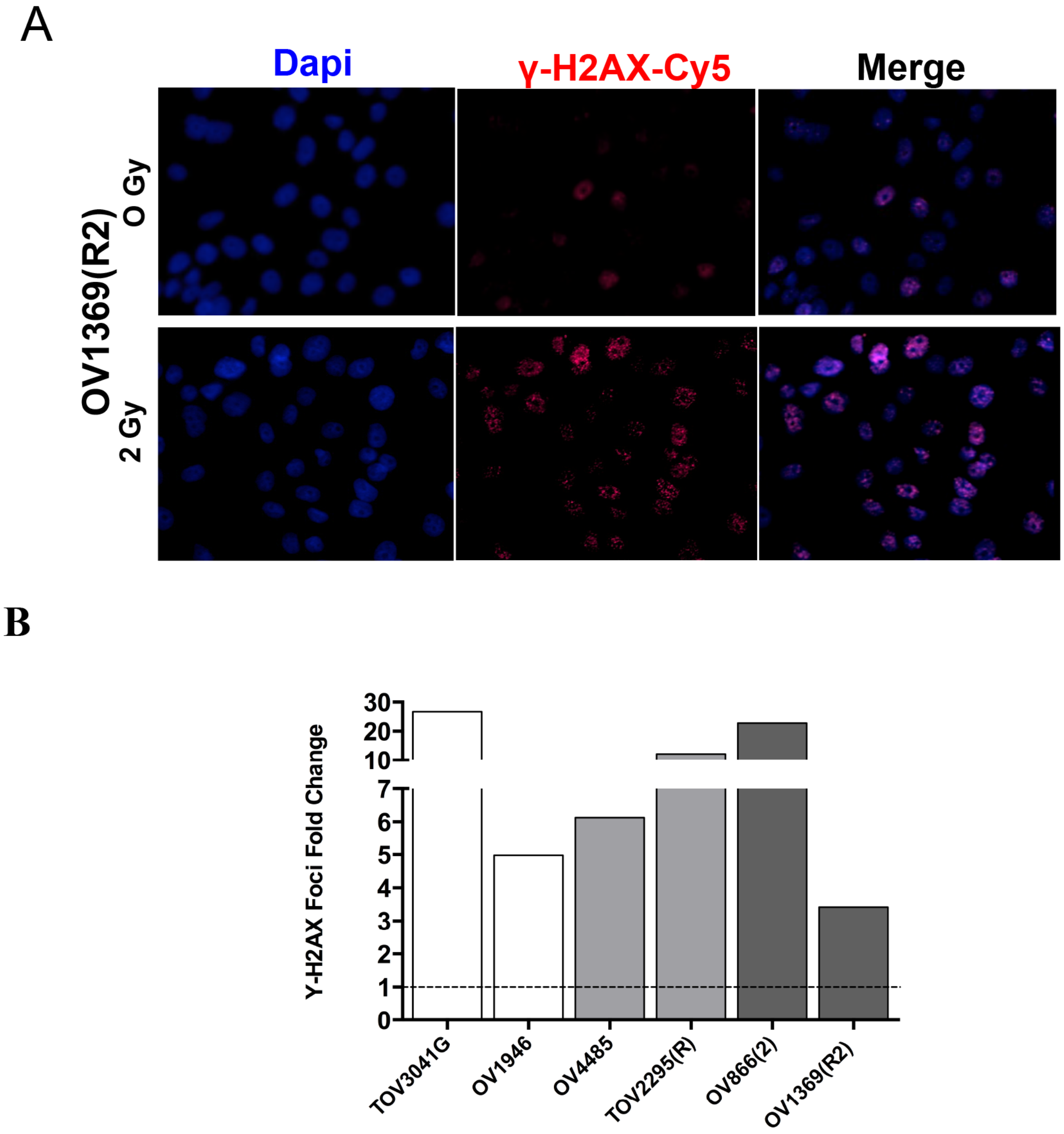

Supplementary Figure S2: Evaluation of γ-H2AX foci formation after gamma-irradiation. A. Representative images of γ-H2AX (red) and nuclear DAPI (blue) staining in resistant OV1369(R2) cell line after 2Gy irradiation. Images are at 400 X magnification. **B.** Evaluation of DNA DSB induction by γ-H2AX immunocytochemistry. Nuclear foci were counted 1 hour after exposure to 2Gy gamma-irradiation in six cell lines of known HR status and Olaparib response. Fold change was calculated as a ratio between percentages of γ-H2AX foci in radiated cells over control non-irradiated cells from one experiment.

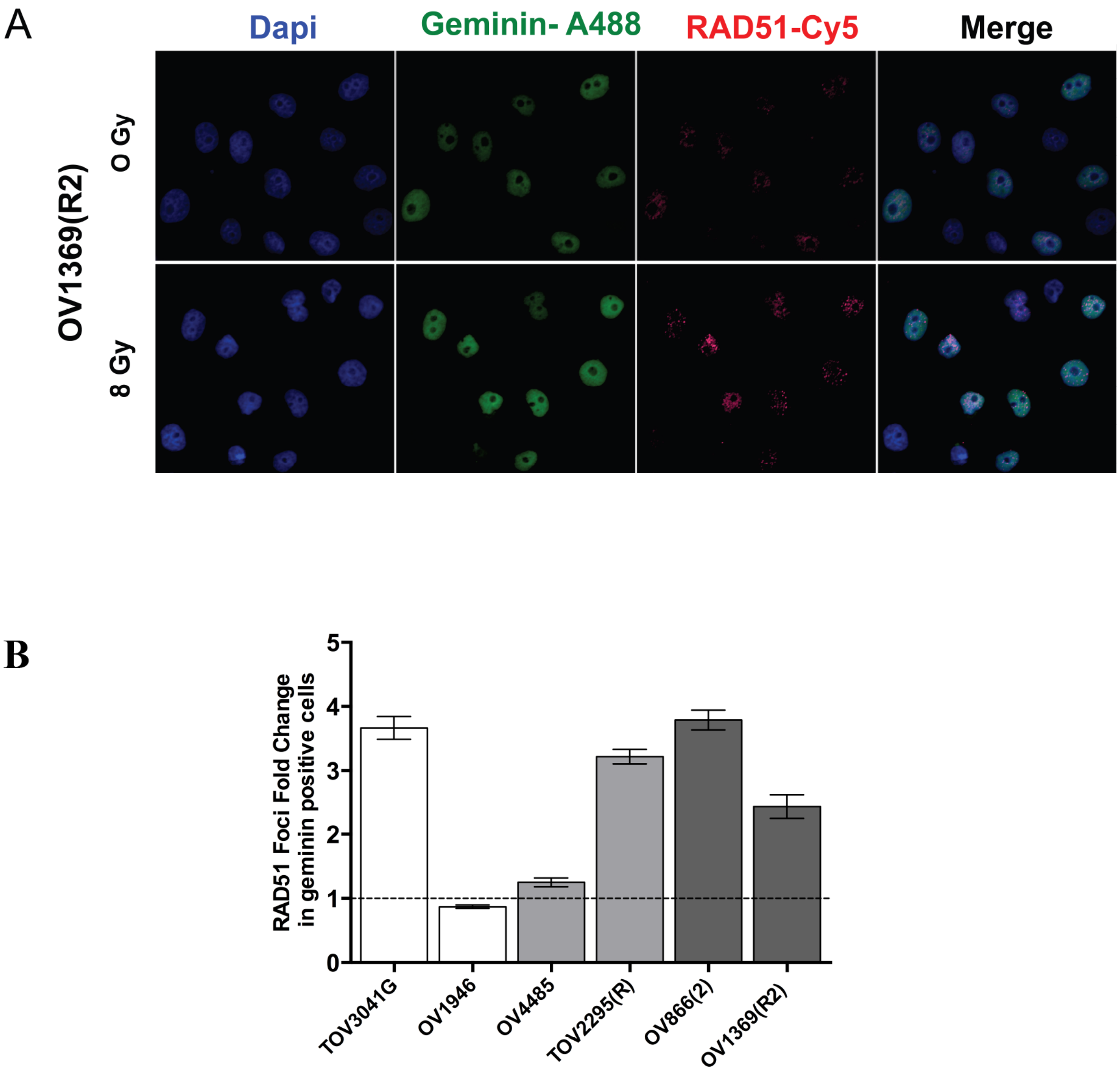

Supplementary Figure S3: Evaluation of RAD51 foci in geminin positive cells. A. Representative images of RAD51 (red) and geminin (green) co-immunostaining, and nuclear DAPI (blue) after 8 Gy irradiation in resistant OV1369(R2) cell line. Images are at 400 X magnification. **B.** Evaluation of HR response by RAD51 immunocytochemistry. Nuclear foci were counted 2 hours after exposure to 8 Gy gamma-radiation, and then compared and presented as percentage of the control group (non-irradiated) in geminin positive cells. Fold change was calculated as a ratio between percentages of RAD51 foci in treated over control non-treated cells. Bars represent average ± SEM from two independent experiments (n=2).

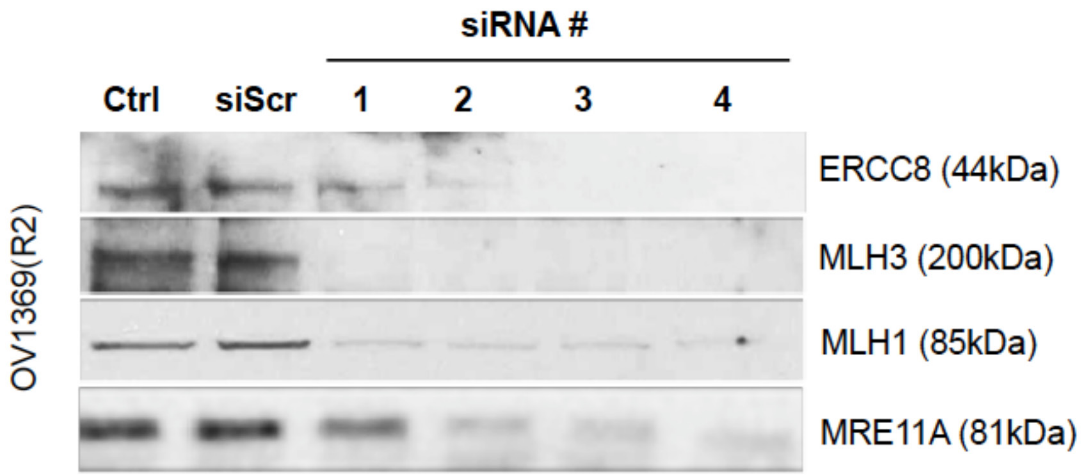

Supplementary Figure S4: Western blots demonstrating decreased protein expression by siRNA knockdowns in resistant cell line OV1369(R2). Wells 1, 2, 3 and 4 correspond to four different siRNA sequences used against each gene (*ERCC8*, *MLH3*, *MLH1* or *MRE11A*). Control = non-treated cells, siScr = negative siRNA control using a scrambled sequence.

A

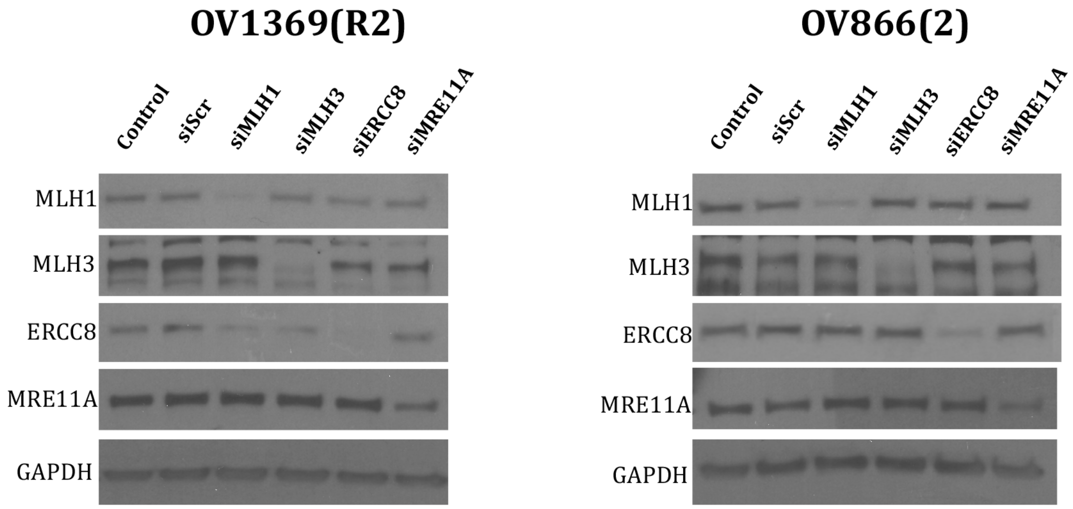

B

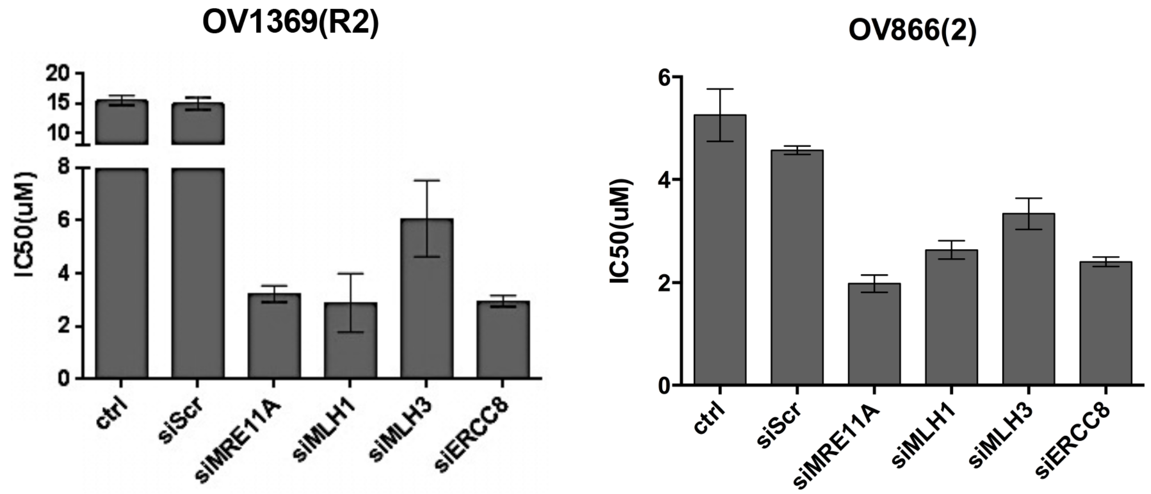

Supplementary Figure S5: Gene knockdown validation using a different siRNA sequence for each target. A. Efficacy of siRNAs against *MLH1*, *MLH3*, *ERCC8* and *MRE11A* in OV1369(R2) and OV866(2) was verified by Western blot. Control = non-treated cells, siScr = negative siRNA control using a scrambled sequence. **B.** Increased Olaparib sensitivity of OV1369(R2) and OV866(2) with siRNAs against *MRE11A*, *MLH1*, *MLH3* and *ERCC8*, assayed by clonogenic assay. Bars represent average ± SEM of IC_{50} values obtained by clonogenic assay in two independent experiments (n=2).

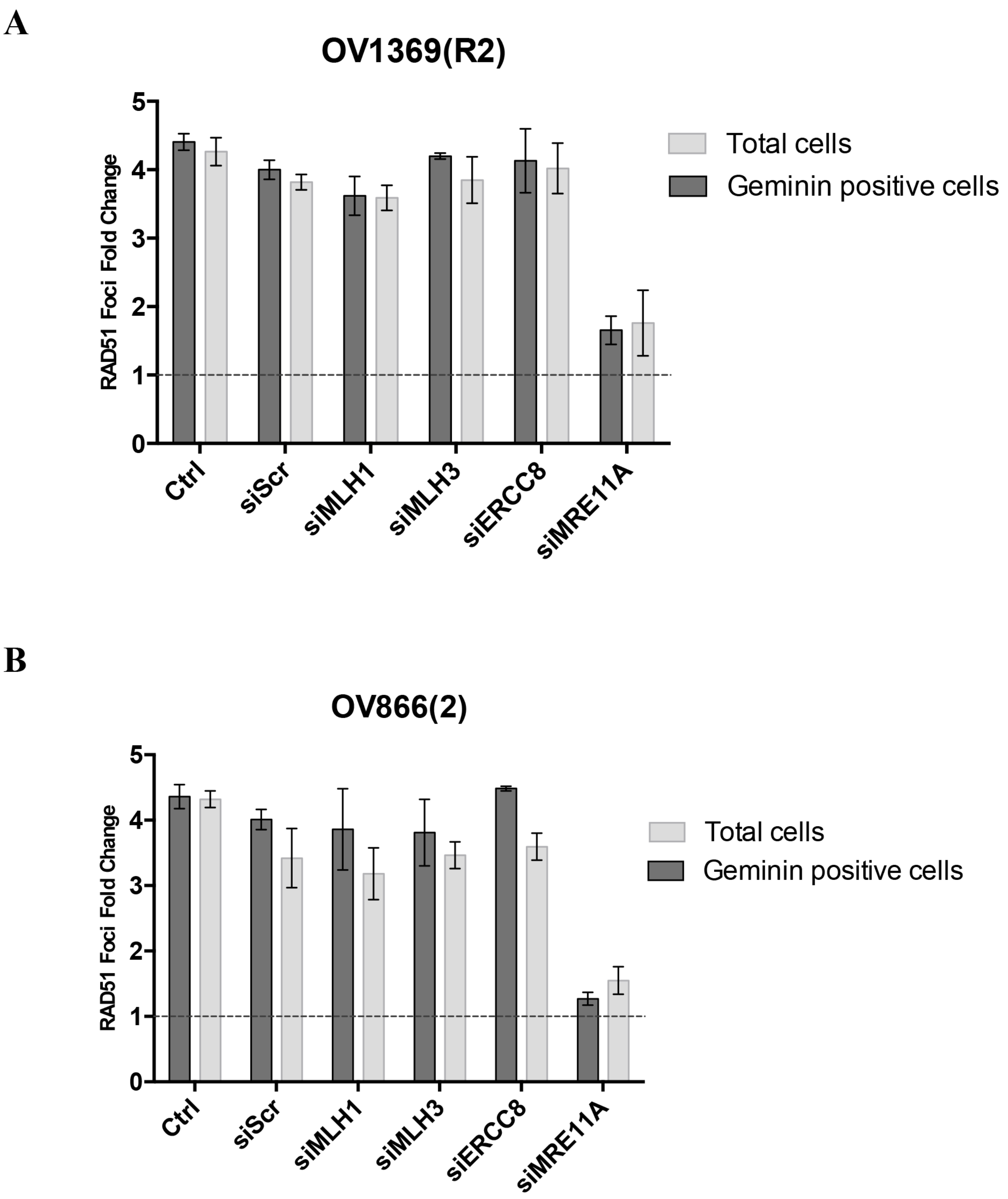

Supplementary Figure S6: HR function after gene knockdown. HR response was evaluated by RAD51 foci analyses in OV1369(R2) **(A)** and OV866(2) **(B)** treated with the same siRNA sequences used in Figures 3 and 4. Nuclear foci were counted 2 hours after exposure to 8 Gy gamma-radiation, and then compared and presented as a percentage of the control group (non-irradiated) in geminin positive (G2/M phase) cells and total cells. Fold change was calculated as a ratio between percentages of RAD51 foci in treated cells over control non-treated cells. Bars represent average ± SEM from two independent experiments (n=2).

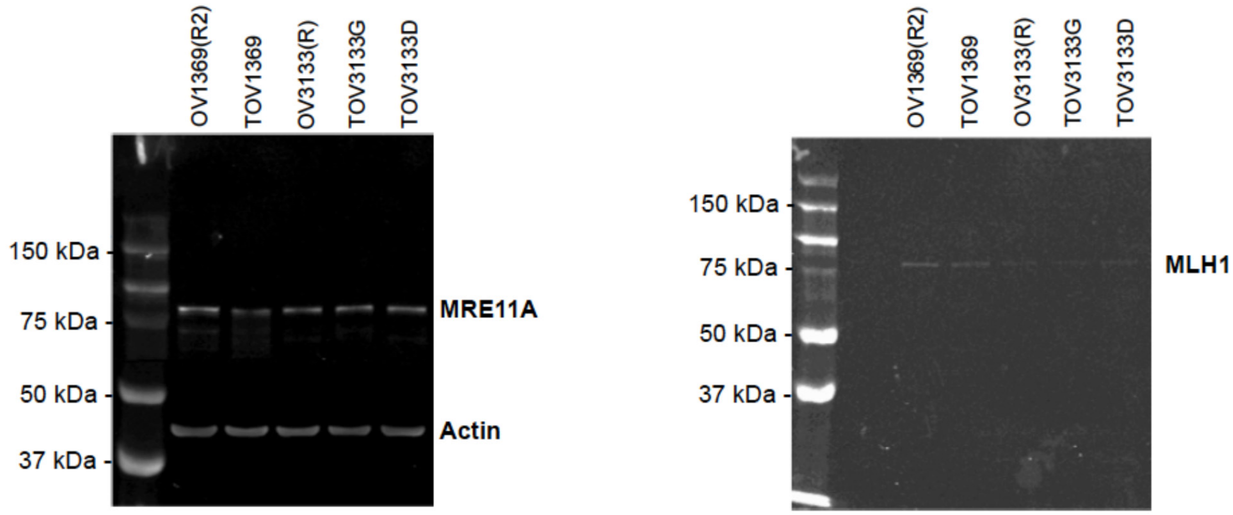

Supplementary Figure S7: Western blot validation of antibodies against MRE11A and MLH1 used for immunohistochemistry analysis of HGS EOC in TMAs. Each well was loaded with 30 µg total cell lysate of HGS EOC cell lines. Actin represents protein loading control.

Supplemnentary Table S1: List of DNA repair genes and Affymetrix probes used in our comparison analysis

Gene	Probesets
APEX1	210027_s_at
APEX2	204408_at
APTX	218527_at
ATM	208442_s_at, 210858_x_at, 212672_at
ATR	209902_at, 209903_s_at
ATRIP	205875_s_at, 34689_at
BLM	205733_at
BRCA1	204531_s_at, 211851_x_at
BRCA2	208368_s_at, 214727_at
BRIP1	221703_at
C19orf40	214816_x_at
CCNH	204093_at
CDK7	211297_s_at
CETN2	209194_at
CHAF1A	203975_s_at, 203976_s_at, 214426_x_at
CHEK1	205393_s_at, 205394_at
CHEK2	210416_s_at
CLK2	203229_s_at
DCLRE1A	209804_at
DCLRE1B	219490_s_at
DCLRE1C	219678_x_at, 222233_s_at
DDB1	208619_at
DDB2	203409_at
DMC1	208382_s_at, 208386_x_at
DUT	208955_at, 208956_x_at, 209932_s_at
ERCC1	203719_at, 203720_s_at
ERCC2	213468_at
ERCC3	202176_at
ERCC4	210158_at
ERCC5	202414_at
ERCC6	207347_at
ERCC8	205162_at
EXO1	204603_at
FANCA	203805_s_at, 203806_s_at, 215530_at

Gene	Probesets
PALB2	219530_at
PARP1	208644_at
PARP2	204752_x_at, 214086_s_at, 215773_x_at
PARP3	209940_at
PCNA	201202_at
PER1	202861_at, 36829_at
PMS1	213677_s_at
PMS2	209805_at, 221206_at
PMS2L3	214473_x_at, 216111_x_at, 216525_x_at
PNKP	218961_s_at
POLB	203616_at
POLD1	203422_at
POLE	216026_s_at
POLG	203366_at, 217635_s_at
POLH	219380_x_at
POLI	219317_at
POLL	221049_s_at
POLM	222238_s_at
POLQ	207746_at, 219510_at
PRKDC	208694_at, 210543_s_at, 215757_at
PRPF19	203103_s_at
PTEN	204053_x_at, 204054_at, 211711_s_at
RAD1	204460_s_at, 204461_x_at, 210216_x_at
RAD17	207405_s_at, 210826_x_at, 211228_s_at
RAD23A	201039_s_at, 201046_s_at
RAD23B	214422_at
RAD23B	201222_s_at, 201223_s_at
RAD50	208393_s_at, 209349_at
RAD51	205023_at, 205024_s_at
RAD51C	206066_s_at, 209849_s_at
RAD51L1	210255_at, 216880_at
RAD51L3	209965_s_at, 37793_r_at
RAD52	205647_at, 210630_s_at, 211904_x_at
RAD54B	220549_at

(*Continued*)

Gene	Probesets
FANCC	205189_s_at
FANCE	220255_at
FANCF	218689_at
FANCG	203564_at
FANCI	213007_at, 213008_at
FANCL	218397_at
FEN1	204767_s_at, 204768_s_at
GIYD1	218317_x_at
GTF2H1	202451_at, 202453_s_at
GTF2H2	221540_x_at
GTF2H3	222104_x_at
GTF2H4	203577_at
GTF2H5	213357_at
H2AFX	205436_s_at, 212524_x_at, 212525_s_at, 213344_s_at
HLTF	202983_at
HUS1	204883_s_at, 204884_s_at, 217618_x_at
LIG1	202726_at
LIG3	204123_at, 207348_s_at
LIG4	206235_at
MBD4	209579_s_at, 209580_s_at, 214047_s_at, 214048_at
MDC1	203061_s_at, 203062_s_at
MGMT	204880_at
MLH1	202520_s_at
MLH3	204838_s_at, 214525_x_at, 217216_x_at
MMS19	202167_s_at
MNAT1	203565_s_at
MPG	203686_at
MRE11A	205395_s_at, 211334_at
MSH2	209421_at
MSH3	205887_x_at, 210947_s_at
MSH4	210533_at
MSH5	210410_s_at, 212913_at
MSH6	202911_at, 211449_at, 211450_s_at
MTMR15	203678_at

Gene	Probesets
RAD54B	219494_at
RAD54L	204558_at
RAD9A	204828_at
RBBP8	203344_s_at
RECQL	205091_x_at, 210568_s_at, 212917_x_at, 212918_at
RECQL4	213520_at
RECQL5	210309_at, 211468_s_at, 221686_s_at, 34063_at
REV1	218428_s_at
REV3L	208070_s_at
RFC1	208021_s_at
RNF4	212696_s_at
RNF8	203160_s_at, 203161_s_at
RPA1	201528_at, 201529_s_at
RPA2	201756_at
RPA3	209507_at
RPA4	221143_at
SETMAR	206554_x_at
SHFM1	202276_at
SMUG1	218685_s_at
TADA3L	215272_at
TDG	203742_s_at
TDG	203743_s_at
TDP1	219715_s_at
TOPBP1	202633_at
TP53	201746_at, 211300_s_at
TP53BP1	203050_at
TREX2	207891_s_at
TTRAP	202266_at
UBE2A	201898_s_at, 201899_s_at
UBE2B	202333_s_at, 202334_s_at, 202335_s_at, 211763_s_at
UBE2N	201523_x_at, 201524_x_at, 212751_at
UBE2V2	209096_at
UNG	202330_s_at
WRN	205667_at

(*Continued*)

Gene	Probesets
MUS81	218463_s_at
MUTYH	207727_s_at
NBN	202905_x_at, 202906_s_at, 202907_s_at, 217299_s_at
NEIL1	219396_s_at
NEIL3	219502_at
NHEJ1	219418_at
NTHL1	209731_at
NUDT1	204766_s_at
OBFC2B	218903_s_at
OGG1	205301_s_at, 205760_s_at

Gene	Probesets
XAB2	218110_at
XPA	205672_at
XPC	209375_at
XRCC1	203655_at
XRCC2	207598_x_at
XRCC3	216299_s_at
XRCC4	205071_x_at, 205072_s_at, 210812_at, 210813_s_at
XRCC5	208642_s_at, 208643_s_at
XRCC6	200792_at, 215308_at

Supplementary Table S2: DNA sequences of siRNAs used in this study

**siERCC8**	1	GUAAAGCAGUGUGUUCCAU
	2	CAGACAAUCUUAUUACACA
	3	CAUCAUAUGUCUCCAGUCU
	4	GAUUGUACUUUAUGACCUU
**siMLH1**	1	GGAAGUUGUUGGAGGUAU
	2	CCAGAUGGUUCGUACAGAU
	3	GAAGUAGUGAUAAGGUCUA
	4	UAUCUUCAUUCUUCGACUA
**siMLH3**	1	CCAAACCAAUCGUCCGUAA
	2	GCUGAGAGCUUAGCAGUUA
	3	ACACAGAGUUCUAGGGAUU
	4	AGACAGGUUUCCAAUGAUA
**siMRE11A**	1	GGAGGUACGUCGUUUCAGA
	2	GGAAAUGAUACGUUUGUAA
	3	CGAAAUGUCACUACUAAGA
	4	GAAAGGCUCUAUCGAAUGU
**siScramble**	1	UCACAACCUCCUAGAAAGAGUAGA
